# Utilization of preconception care and its impacts on health behavior changes among expectant couples in Shanghai, China

**DOI:** 10.1186/s12884-021-03940-0

**Published:** 2021-07-07

**Authors:** Li Du, Xuena La, Liping Zhu, Hong Jiang, Biao Xu, An Chen, Mu Li

**Affiliations:** 1 Shanghai Center for Women and Children’s Health, 339 Luding Road, Putuo District, Shanghai, 200062 China; 2 School of Public Health; Global Health Institute; Key Lab of Health Technology Assessment, National Health Commission of the People’s Republic of China, Fudan University, Mailbox 175, No. 138 Yixueyuan Road, Shanghai, 200032 China; 3 Shanghai Municipal Center for Disease Control and Prevention, No.1380 West Zhongshan Road, Shanghai, 200336 China; 4 Institute of Healthcare Engineering, Management and Architecture (HEMA Institute), Department of Industrial Engineering and Management, Aalto University, Maarintie 8, FI-02150 Espoo, Finland; 5 School of Public Health, China Studies Centre, University of Sydney, Room 313, Edward Ford Building, Sydney, 2006 Australia

**Keywords:** Preconception care, Health behavior change, Smoking, Drinking, Folic acid supplements

## Abstract

**Background:**

Preconception care is an opportunity for detecting potential health risks in future parents and providing health behavior education to reduce morbidity and mortality for women and their offspring. Preconception care has been established in maternal and child health hospitals in Shanghai, China, which consists of health checkups, health education and counseling. This study investigated factors associated with the utilization of preconception care, and the role of preconception care on health behavior changes before conception among pregnant women and their partners.

**Methods:**

A cross-sectional study was conducted among pregnant women at three maternal and child health hospitals in Shanghai. The participants were invited to complete a self-administered questionnaire on the utilization of preconception care and health behavioral changes before conception.

**Results:**

Of the 948 recruited pregnant women, less than half (42.2%) reported that they had utilized preconception care before the current pregnancy. Unplanned pregnancy, unawareness of preconception care and already having a general physical examination were the main reasons for not attending preconception care. The two main sources of information about preconception care were local community workers and health professionals. Younger women and the multipara were less likely to utilize preconception care. Women who utilized preconception care were more likely to take folic acid supplements before conception [Adjusted Odds Ration (aOR) 3.27, 95% Confidence Interval (CI) 2.45–4.36, *P* < 0.0001]. The partners of pregnant women who had attended preconception care services were more likely to stop smoking [aOR 2.76, 95%CI 1.48–5.17, *P* = 0.002] and to stop drinking [aOR 2.13, 95%CI 1.03–4.39, *P* = 0.041] before conception.

**Conclusions:**

Utilization of preconception care was demonstrated to be positively associated with preconception health behavior changes such as women taking folic acid supplements before pregnancy, their male partner stopping smoking and drinking before conception. Future studies are needed to explore barriers to utilizing preconception care services and understand the quality of the services. Strategies of promoting preconception care to expectant couples, especially to young and multipara women, should be developed to further improve the utilization of the services at the community level.

## Background

The Developmental Origins of Health and Disease (DOHaD) concept suggests that early life exposures to maternal, perinatal, and early childhood risk factors can affect fetal and child growth and development that have long-term consequences on later health [1]. The critical period for fetal development occurs before many women even aware of the conception. The first contact with antenatal care is often too late for preventing or reducing adverse pregnancy outcomes such as congenital malformations, preterm birth, and low birth weight [2]. Promoting preconception health care is not only an important public health strategy to improve women’s health and pregnancy outcomes, but also to protect a child’s health, growth, and development.

Preconception unhealthy behaviors of parents are associated with increased risks of morbidity and mortality of offspring. For example, excessive alcohol consumption of women before pregnancy is associated with premature rupture of membrane, lower birth weight, spontaneous abortions, and significantly lower IQ in preschool children [3–5]. Men’s health also plays an important role in pregnancy and health outcomes of future generations through the direct and genetic influence of spermatozoa quality [6, 7]. Growing evidence shows that changing health-related behaviors before conception can have a positive effect on improving pregnancy outcomes. Women taking folic acid supplements before pregnancy could prevent neural tube disorders (NTDs) in the offspring [8, 9]. Mothers who stopped smoking before pregnancy could benefit fetal and child growth and development [10, 11].

The World Health Organization (WHO) recommends the provision of preconception care (PCC) as a way to increase the health and well-being of women and couples, and improve subsequent pregnancy and child health outcomes [12]. To prevent and reduce birth defects, in 2007 the Ministry of Health of China released the National Guidelines for Preconception Care, and in 2010 the Chinese National Health and Family Planning Commission and Ministry of Finance formally launched the National Free Preconception Health Examination Project (NFPHEP) to support preconception health services in selected rural counties. The initiative was expanded covering all rural childbearing-age couples nationwide in 2013 [13]. It was encouraged that the initiative is implemented in urban areas as well.

Since 2013, PCC services, funded by the Shanghai Municipal Government, have been available to all couples of childbearing-age in Shanghai. Couples living in a district of Shanghai with at least one person who has permanent residence registration can apply for free PCC services at the district maternal and child health hospital/institute. The PCC services provided in Shanghai are based on China’s Recommendations for Preconception and Prenatal Care [14]. The preconception care services consist of 1) medical history, 2) clinical examination of the reproductive tract, 3) laboratory tests, including blood tests, urinary analysis and serology tests, and 4) medical consultation and counseling for the couple according to the examination and laboratory test results, and health education on pregnancy spacing, preconception nutrition, environmental exposure, healthy preconception lifestyle e.g. ceasing of smoking and drinking, and timely prenatal care utilization [14].

Although preconception care services have been implemented for several years in Shanghai, the participation of PCC and the impact of the services, including the effects on health behavioral change, have rarely been evaluated. To analyze the association between utilization of PCC services and health behavior change will help to understand the policy impact and provide evidence for future service improvement. The purpose of this study was to examine factors associated with the utilization of PCC services, and to assess the associations between the utilization of PCC services and several preconception behavior changes, including cessation of smoking and drinking before conception of couples, avoidance of secondhand smoke and intake of folic acid (FA) supplements during preconception among women. We hypothesized that positive preconception behavior changes such as cessation of drinking and smoking in both women and their partners, avoidance of secondhand smoke and FA supplements intake among women before conception were associated with utilization of PCC services.

## Methods

### Study design

This was a cross-sectional survey conducted at three maternal and child health (MCH) hospitals in three districts of Shanghai, an urban district, a suburban district, and an outer suburban district, between July and August 2017. Each of them is the only MCH hospital in the district, therefore, most annual births in these districts occurred in these hospitals. Each hospital was required to recruit at least 300 pregnant women, and a convenient sampling method was used. Pregnant women were approached and invited by researchers to complete the questionnaire if they were eligible for participating in the survey during their visits to antenatal clinics in the study period. The inclusion criteria were pregnant women who were willing to provide a written informed consent and to spend about 15 min to complete the questionnaire. The exclusion criteria were women who had cognitive impairment and were unable to complete the questionnaire independently. All participants provided written informed consent.

### Measurements

The study used a purposely designed questionnaire, consisting of two sections. The first section collected demographic characteristics information, including age, gestational age, household registration, education level, family income per month, employment status and parity. The second section focused on the utilization of PCC services and health-related behavior changes. The questionnaire was developed based on literature review, professional experience, and finalized after a group of expert consultations. We performed a pilot test with 20 pregnant women and improved the questionnaire based on the pilot test results before used in the study.

The exposure variable of this study was the utilization of PCC services. The outcome variables were preconception behavior changes, including smoking cessation and drinking cessation prior to pregnancy in both pregnant women and their partners, avoidance of secondhand smoke exposure and intake of FA supplements during preconception among the women.

All participants were asked whether they had attended PCC services before the current pregnancy. Participants who answered “yes” were asked the main information sources, and those who answered “no” were inquired about the reasons for not attending the PCC services. Preconception tobacco consumption was assessed by the question “Do you smoke?” Among women who answered “yes”, further two questions were asked “How many cigarettes do you smoke each day on average?” and “In the 3 months before you got pregnant, did you stop smoking?” The consumption and cessation of alcohol and tobacco in both women and their partners were assessed by similar questions. Any tobacco and alcohol use before pregnancy were coded as preconception tobacco use and preconception alcohol use. Women’s exposure to secondhand smoke during preconception and whether they had avoided secondhand smoke exposure in the 3 months before being pregnant were also assessed. Preconception intake of FA supplementation was assessed by the question, “Did you take folic acid supplement before conception?” Responses were “yes” or “no”.

### Data analysis

We used descriptive analysis (percentage) to report women’s utilization of PCC services and health-related behaviors. We analyzed the factors associated with the utilization of PCC services by computing the prevalence Odds Ratios (ORs) and 95% Confidence Intervals (95% CIs). We examined associations between the utilization of PCC services and behavior changes using multivariable logistic regressions with each behavior change as the dependent variable and the utilization of PCC services as an independent variable. We considered potential confounders such as women’s age, household registration, family income, employment status, and parity in the multiple regression models [15–17]. Adjusted Odds Ratio (aOR) and 95% CI were used for interpretation of the adjusted risk and *p* < 0.05 was considered statistically significant. Statistical analyses were carried out using SAS 9.4.

## Results

### Demographic characteristics of the participants

A total of 983 pregnant women participated in the survey and 948 completed questionnaires were valid for data analysis. The mean age of the pregnant women was 29 years, ranged from 15 to 45 years. More than half of them (56.3%, 534/948) were below 30 years old. The average gestational age was 25 weeks, ranging between 3 and 41 weeks. Only 32.7% of women (310/948) had Shanghai permanent residency registration. Most women (78.3%,742/948) had college or above education and were employed (76.4%, 724/948). The majority of women’s (91.9%, 871/948) monthly family income was above 5000RMB (~713USD, double the amount of the minimum monthly income of Shanghai). Nearly 70% of pregnant women (68.9%, 653/948) were primiparas (Table 1).

**Table 1:** Demographic characteristics of participants (*N* = 948)

Characteristic	Overall		Women participated in PCC (***n*** = 400)		Women not participated in PCC (***n*** = 548)		cOR (95% CI)	aOR (95% CI)	***P***
***Gestational age (week, Mean ± SD)***	25.74 ± 8.43		26.39 ± 8.41		25.27 ± 8.43		–	–	–
	**N**	**%**	**N**	**%**	**N**	**%**			
***Age***									
< 30 years	534	56.3	203	50.8	331	60.4	ref	ref	
≥ 30 years	414	43.7	197	49.2	217	39.6	1.48 (1.14,1.92)	1.85 (1.37,2.52)	< 0.001
***Household registration***									
Shanghai	310	32.7	146	36.5	164	29.9	ref	ref	
Non-Shanghai	638	67.3	254	63.5	384	70.1	0.74 (0.57,0.98)	0.82 (0.61,1.10)	0.192
***Education level***									
Junior middle school and below	85	9.0	29	7.3	56	10.2	ref	ref	
Senior middle school	121	12.7	50	12.5	71	13.0	1.36 (0.77,2.43)	1.42 (0.78,2.61)	0.256
College and above	742	78.3	321	80.2	421	76.8	1.47 (0.93,2.39)	1.35 (0.77,2.39)	0.294
***Family income per month***									
< 5000RMB	77	8.1	30	7.5	47	8.6	ref	ref	
5000 – 10000RMB	264	27.9	113	28.2	151	27.5	1.17 (0.70,1.98)	1.05 (0.61,1.82)	0.873
≥ 10000RMB	607	64.0	257	64.3	350	63.9	1.15 (0.71,1.89)	0.92 (0.53,1.61)	0.774
***Employment status***									
Employed	724	76.4	302	75.5	422	77.0	ref	ref	
Unemployed	224	23.6	98	24.5	126	23.0	1.09 (0.80,1.47)	1.37 (0.97,1.95)	0.075
***Parity***									
0	653	68.9	291	72.8	362	66.1	ref	ref	
≥ 1	295	31.1	109	27.3	186	33.9	0.72 (0.55,0.97)	0.56 (0.41,0.77)	< 0.001
***Preconception tobacco use***									
No	925	97.6	393	98.3	532	97.1	ref	ref	
Yes	23	2.4	7	1.7	16	2.9	0.59 (0.23,1.40)	0.60 (0.22,1.49)	0.288
***Preconception Secondhand smoke exposure***									
No	775	81.8	328	82.0	447	81.6	ref	ref	
Yes	173	18.2	72	18.0	101	18.4	0.97 (0.69,1.35)	1.06 (0.74,1.51)	0.769
***Preconception alcohol use***									
No	889	93.8	379	94.8	510	93.1	ref	ref	
Yes	59	6.2	21	5.2	38	6.9	0.74 (0.42,1.28)	0.72 (0.39,1.29)	0.274

*PCC* Preconception Care, *cOR* crude odds ratio, *aOR* adjusted odds ratio

### The utilization of PCC services and associated factors

Less than 50% (42.2%, 400/948) of pregnant women reported they had utilized PCC services before the current conception. The top three reasons for not attending PCC were unplanned pregnancy (34.3%, 188/548), unawareness of the PCC (22.2%, 122/548), and already had a physical examination (18.6%, 102/548). The proportion of unplanned pregnancies was high in this study, almost half of the pregnancies (47.4%, 449/948) were not planned. The sources of information about PCC services among those who attended PCC were local community workers (40.0%, 160/400), health professionals (33.0%, 132/400), and friends and family members (17.5%, 70/400). Age and parity were associated with the utilization of PCC. Women who were 30 years old and above were more likely to participate in PCC compared with women younger than 30 years old (aOR 1.85, 95%CI 1.37–2.52, *P* < 0.001). Compared with primiparas, the multipara were less likely to utilize PCC services (aOR 0.56, 95%CI 0.41–0.77, *P* < 0.001) (Table 1).

### Association between the utilization of PCC services and health behavior changes before conception

Most pregnant women (97.6%, 925/948) reported that they had never smoked before pregnancy. Among the 23 smokers, 9 (39.1%) stated that they stopped smoking when preparing for pregnancy. Nearly 20% of women (173/948) reported they were exposed to secondhand smoke before preparing for pregnancy, and 67.1% of them (116/173) avoided exposure to secondhand smoke before being pregnant. The Fisher’s exact and Chi-square test showed neither cessation of smoking (χ^2^ = 0.059, *P* = 1.000) nor avoidance of secondhand smoke exposure (χ^2^ = 0.340, *P* = 0.560) before conception among women was significantly associated with the utilization of PCC services. Multivariable logistic regression results showed that the utilization of PCC services was not significantly associated with avoidance of secondhand smoke exposure before conception among women (aOR 0.75, 95% CI 0.38–1.49, *P* = 0.410) (Table 2).

**Table 2:** Association between avoidance of secondhand smoke exposure /cessation of drinking and PCC services utilization before conception among pregnant women

Avoidance of secondhand smoke exposure before conception(***N*** = 173^a^)					Drinking cessation before conception(***N*** = 59^a^)			
	n(%)^b^	cOR (95% CI)	aOR(95% CI)^c^	*P*	n(%)^b^	cOR (95% CI)	aOR (95% CI) ^c^	*P*
**Utilization of PCC services**								
Yes	46 (63.89)	0.78 (0.41, 1.49)	0.75 (0.38, 1.49)	0.410	13 (61.90)	2.01 (0.68, 5.96)	1.43 (0.38, 5.34)	0.592
No	70 (69.31)	1.00	1.00		17 (44.74)	1.00	1.00	

*PCC* Preconception Care, *cOR* crude odds ratio, *aOR* adjusted odds ratio

^a^ Logistic regression was performed among women who reported they were exposed to secondhand smoke (*N* = 173)/drank(*N* = 59) before pregnancy

^b^ Unweighted n, weighted percent

^c^ Adjusted for education level, family income per month, age, household registration, employment status, parity

Only 6.2% (59/948) of women revealed they ever drank alcohol before conception, and half of them (50.8%, 30/59) stopped drinking before pregnancy. The Chi-square test (χ^2^ = 0.982, *P* = 0.322) did not show statistically significant association between women stopping drinking and the utilization of PCC services. Multivariable logistic regression results showed there was no association between the utilization of PCC services and drinking cessation before conception among pregnant women (aOR 1.43, 95% CI 0.38–5.34, *P* = 0.592) (Table 2).

Nearly 30% of women (29.2%, 277/948) reported their partners were smokers before conception. Among those men, 21.3% (59/277) stopped smoking when preparing for pregnancy. Among 155 male partners who ever drank alcohol before considering pregnancy, 35.7% (55/155) of them stopped drinking before conception. Multivariable logistic regression results showed that partners of pregnant women who utilized PCC services were more likely to stop smoking (aOR 2.76, 95%CI 1.48–5.17, *P* = 0.002), and to stop drinking (aOR 2.13, 95%CI 1.03–4.39, *P* = 0.041) before conception (Table 3).

**Table 3:** Association between cessation of smoking, drinking before conception and PCC services utilization among partners of pregnant women

	Smoking cessation before conception(***N*** = 277^a^)				Drinking cessation before conception(***N*** = 155^a^)			
	n(%)^b^	cOR (95% CI)	aOR(95% CI)^c^	*P*	n(%)^b^	cOR (95% CI)	aOR (95% CI) ^c^	*P*
**Utilization of PCC services**								
Yes	32 (30.28)	2.35 (1.31, 4.22)	2.76 (1.48, 5.17)	0.002	29 (43.94)	1.90 (0.98, 3.70)	2.13 (1.03, 4.39)	0.041
No	27 (15.70)	1.00	1.00		26 (29.21)	1.00	1.00	

*PCC* Preconception Care, *cOR* crude odds ratio, *aOR* adjusted odds ratio

^a^ Logistic regression was performed among women who reported their partners smoked(*N* = 277)/drank(*N* = 155) before pregnancy

^b^ Unweighted n, weighted percent

^c^ Adjusted for education level, family income per month, age, household registration, employment status, parity

More than half (59.0%, 559/948) of pregnant women reported that they took FA supplements before conception. Multivariable logistic regression results showed women who utilized PCC services were more likely to start taking FA supplements before conception (aOR 3.27, 95%CI 2.45–4.36, *P* < 0.0001) compared with those who did not attend PCC services (Table 4).

**Table 4:** Association between preconception intake of folic acid supplements and PCC services utilization among pregnant women

	Taking folic acid supplements before conception(***N*** = 948)			
	n(%)^a^	cOR (95% CI)	aOR (95% CI)^b^	*P*
**Utilization of PCC services**				
Yes	300 (75.00)	3.35 (2.53, 4.44)	3.27 (2.45, 4.36)	< 0.0001
No	259 (47.26)	1.00	1.00	

*PCC* Preconception Care, *cOR* crude odds ratio, *aOR* adjusted odds ratio

^a^ Unweighted n, weighted percent

^b^ Adjusted for education level, family income per month, age, household registration, employment status, parity

## Discussion

Our study showed that despite the benefits and wide availability of PCC services in Shanghai since 2013, only 42.2% of pregnant women utilized PCC services. Younger women and the multipara had used PCC services less than other groups. We found that women who had utilized PCC services were more likely to start taking folic acid before conception, and their partners were more likely to stop smoking and drinking before conception.

Just over 40 % of pregnant women in this study reported that they ever attended PCC services, which was similar to the rate of PCC services utilization reported in other areas of China [18–20]. Unplanned pregnancy was identified as one of the main reasons for not using PCC services suggesting that couples lacked the awareness of preparing for the pregnancy [21]. The prevalence of unplanned pregnancy was as high as 47.5% in our study. Unintended and unplanned pregnancies were associated with induced abortion, delayed initiation of antenatal care, and unhealthy behavior during pregnancy [22–24]. Women who previously had a full physical examination did not think they would need PCC suggesting the lack of comprehensive understanding of the importance of PCC among some women of reproductive age. Strategies for improving the awareness of the importance of pregnancy planning and the benefits of PCC among reproductive-age couples need to be further explored. Local community workers and health professionals were found to be the main information sources of PCC services among participants. This reflected the fact that the PCC service system in China was carried out based on the primary health and planned parenthood network [13]. Local community staff are responsible for identifying couples with intention of conception and recommending them to utilize the free PCC services at the district maternal and child health hospitals/institute [25]. Public awareness and PCC services promotion at the community level should be strengthened.

A higher rate of utilization of PCC services was found among the women older than 30 years and primipara. This finding is in line with other studies examining the factors associated with the utilization of PCC services [15, 16, 26]. This is perhaps because older women were more concerned about the conception and positive experience of pregnancy. Multiparas tended to use PCC services less, this might be due to that they could draw on their previous experiences of pregnancy and childbirth, and nurturing a healthy baby. There might also be a misconception that the PPC was especially for couples expecting their first child. A similar finding was reported by You et al., that families with more than one child had a low rate of PCC services utilization [15]. Future PCC promotion efforts should be focused on younger and multipara segments of the target population.

Our study documented that women who utilized PCC services were more likely to start taking FA before conception. Similar studies have shown the improvement in knowledge and behavior of FA supplementation in women who utilized PCC services before conception [27, 28]. The WHO recommends that all women should take 400 μg FA supplements daily when they begin trying to conceive until 12 weeks of gestation [29]. The current Recommendations for the Preconception and Prenatal Care of China suggest women with the intake of 400 μg FA per day from three months before conception to the end of the first trimester of pregnancy [14]. Our findings advocate for the universal coverage of PCC to promote FA supplementation before conception among women of reproductive age.

 We have captured that partners of women who attended the PCC were more likely to stop smoking and drinking before conception. This finding suggested that involving men in the PCC has the benifit of influencing their health behavior change. The positive association between the utilization of PCC services and health behavioral changes including smoking and drinking cessation among male partners before conception was consistent with findings of other studies [16, 30]. Jill Shawe et al. found that preconception health education by general practitioners or health professionals was significantly associated with reduced smoking and drinking among the men planning for pregnancy [30]. Preconception care for men has the potential to improve family planning and pregnancy outcomes, enhance the health behaviors of their female partners, and prepare for fatherhood [6, 7, 31]. However, a recent systematic review exploring preconception health behaviors found that only 11% of studies included men, which indicated that little attention had been given to men’s preconception care [32]. A study including 566,439 preconception fathers found that compared to those who continued smoking from preconception to early pregnancy, fathers who quitted or decreased smoking during preconception were at lower risk of having a child with birth defects [33].

Our results did not show any association between the utilization of PCC services and cessation of drinking and smoking in women, which is inconsistent with findings from other studies [34, 35]. This was likely due to the inadequate statistical power to detect the difference resulted from the very low self-reported rate of smoking and drinking among the Chinese women of this study. The rates of drinking and smoking are generally lower among Chinese women compared with women in western countries [36, 37].

The study has several limitations. First, the health behavior changes we investigated were limited to smoking and drinking cessation, and intake of folic acid supplements. Second, information about behavior changes of male partners was reported by women indirectly, which could lead to bias on the estimates of alcohol and tobacco consumption. Third, this was a cross-sectional study which was not an ideal study design to determine the role of the PCC in changing harmful behaviors and medical conditions. Fourth, the non-random sampling process might lead to selection bias. Fifth, we did not conduct a full psychometric test of the questionnaire. Finally, we did not follow the women to childbirth, therefore, the pregnancy and birth outcomes were not included in the analysis. Future qualitative studies are needed to determine barriers to utilizing preconception care services and understand the quality of the services. A prospective cohort study will help to determine the causal relationship between utilization of PCC services and behavior changes, and explore the reasons for behavior changes and non-behavior changes.

## Conclusion

Only around 40 % of pregnant women in this study reportedly utilized PCC services. Unplanned pregnancy, lack of awareness for the PCC services, and having undergone a general physical examination were identified as the main reasons for not attending PCC services. Our study focused on the role of PCC services on the changes of several health behaviors among preconception couples, which had rarely been evaluated. The findings highlighted the importance of PCC services in providing the opportunity for couples to improve health-related behaviors before pregnancy. Strategies of promoting preconception care to expectant couples especially among the younger and the multipara women at the community level should be developed to further improve the utilization of the services. Future studies are needed to explore barriers to utilizing preconception care services and evaluate the quality of PCC services.

## Data Availability

The datasets used and/or analyzed during the current study are available from the corresponding author on reasonable request.

## Abbreviations

DOHaD: Developmental Origins of Health and Disease
NTDs: Neural Tube Disorders
WHO: World Health Organization
PCC: Preconception Care
NFPHEP: National Free Preconception Health Examination Project
FA: Folic Acid
MCH: Maternal and Child health
